# Supplementary material for: Modeling glioblastoma heterogeneity as a dynamic network of cell states
Source: Mol Syst Biol. 2021 Sep 16;17(9):e10105. doi: 10.15252/msb.202010105 (PMC8444284; doi:10.15252/msb.202010105)
Supplement: Supplementary file 5 — Source Data for Figure 3 [file MSB-17-e10105-s001.zip › Figure3A_sourcedata/GSEA_3065/hallmarks_state4.GseaPreranked.1623416296068/HALLMARK_GLYCOLYSIS.html]

Details for gene set HALLMARK\_GLYCOLYSIS[GSEA]

|  || Dataset | state4 |
| Phenotype | NoPhenotypeAvailable |
| Upregulated in class | na\_neg |
| GeneSet | HALLMARK\_GLYCOLYSIS |
| Enrichment Score (ES) | -0.37276644 |
| Normalized Enrichment Score (NES) | -1.354298 |
| Nominal p-value | 0.047557842 |
| FDR q-value | 0.14677261 |
| FWER p-Value | 0.906 |
Table: GSEA Results Summary

  

Fig 1: Enrichment plot: HALLMARK\_GLYCOLYSIS      
 Profile of the Running ES Score & Positions of GeneSet Members on the Rank Ordered List

  

| PROBE | GENE SYMBOL | GENE\_TITLE | RANK IN GENE LIST | RANK METRIC SCORE | RUNNING ES | CORE ENRICHMENT || 1 | IRS2 |  |  | 33 | 0.338 | 0.0255 | No |
| 2 | SOX9 |  |  | 48 | 0.299 | 0.0505 | No |
| 3 | PGLS |  |  | 190 | 0.206 | 0.0464 | No |
| 4 | GNPDA1 |  |  | 247 | 0.186 | 0.0544 | No |
| 5 | GALK1 |  |  | 381 | 0.157 | 0.0471 | No |
| 6 | SOD1 |  |  | 396 | 0.152 | 0.0587 | No |
| 7 | DDIT4 |  |  | 493 | 0.138 | 0.0558 | No |
| 8 | PPP2CB |  |  | 588 | 0.127 | 0.0521 | No |
| 9 | FAM162A |  |  | 660 | 0.119 | 0.0514 | No |
| 10 | CHPF |  |  | 706 | 0.113 | 0.0544 | No |
| 11 | HDLBP |  |  | 827 | 0.102 | 0.0442 | No |
| 12 | TALDO1 |  |  | 830 | 0.101 | 0.0531 | No |
| 13 | ARPP19 |  |  | 843 | 0.100 | 0.0603 | No |
| 14 | SDHC |  |  | 880 | 0.097 | 0.0633 | No |
| 15 | PAM |  |  | 903 | 0.096 | 0.0685 | No |
| 16 | RRAGD |  |  | 1070 | 0.083 | 0.0491 | No |
| 17 | MIF |  |  | 1100 | 0.082 | 0.0519 | No |
| 18 | MDH2 |  |  | 1114 | 0.081 | 0.0572 | No |
| 19 | ALDH7A1 |  |  | 1172 | 0.078 | 0.0550 | No |
| 20 | MXI1 |  |  | 1247 | 0.073 | 0.0497 | No |
| 21 | NOL3 |  |  | 1274 | 0.072 | 0.0521 | No |
| 22 | PYGL |  |  | 1349 | 0.069 | 0.0463 | No |
| 23 | TPST1 |  |  | 1469 | 0.063 | 0.0327 | No |
| 24 | IDH1 |  |  | 1691 | 0.055 | 0.0019 | No |
| 25 | B4GALT7 |  |  | 1890 | 0.050 | -0.0258 | No |
| 26 | DLD |  |  | 1906 | 0.049 | -0.0237 | No |
| 27 | VLDLR |  |  | 1952 | 0.048 | -0.0266 | No |
| 28 | B4GALT2 |  |  | 1975 | 0.048 | -0.0259 | No |
| 29 | STC1 |  |  | 2141 | 0.043 | -0.0488 | No |
| 30 | SDC3 |  |  | 2214 | 0.041 | -0.0567 | No |
| 31 | DPYSL4 |  |  | 2355 | 0.038 | -0.0760 | No |
| 32 | AGL |  |  | 2452 | 0.036 | -0.0883 | No |
| 33 | AK3 |  |  | 2544 | 0.034 | -0.1000 | No |
| 34 | CAPN5 |  |  | 2654 | 0.032 | -0.1148 | No |
| 35 | CD44 |  |  | 2687 | 0.031 | -0.1172 | No |
| 36 | UGP2 |  |  | 2722 | 0.030 | -0.1200 | No |
| 37 | CASP6 |  |  | 2748 | 0.030 | -0.1213 | No |
| 38 | IL13RA1 |  |  | 2751 | 0.030 | -0.1189 | No |
| 39 | BPNT1 |  |  | 2756 | 0.030 | -0.1169 | No |
| 40 | PPIA |  |  | 2777 | 0.029 | -0.1175 | No |
| 41 | MPI |  |  | 2832 | 0.028 | -0.1238 | No |
| 42 | PGK1 |  |  | 3012 | 0.025 | -0.1506 | No |
| 43 | MED24 |  |  | 3182 | 0.021 | -0.1761 | No |
| 44 | NDUFV3 |  |  | 3215 | 0.021 | -0.1794 | No |
| 45 | GCLC |  |  | 3344 | 0.017 | -0.1987 | No |
| 46 | GPC4 |  |  | 3380 | 0.016 | -0.2029 | No |
| 47 | TPI1 |  |  | 3442 | 0.014 | -0.2115 | No |
| 48 | ZNF292 |  |  | 3453 | 0.014 | -0.2119 | No |
| 49 | COPB2 |  |  | 3552 | 0.011 | -0.2269 | No |
| 50 | MDH1 |  |  | 3601 | 0.009 | -0.2339 | No |
| 51 | G6PD |  |  | 3603 | 0.009 | -0.2332 | No |
| 52 | B3GAT1 |  |  | 3752 | 0.003 | -0.2570 | No |
| 53 | VEGFA |  |  | 3787 | 0.001 | -0.2624 | No |
| 54 | AGRN |  |  | 3832 | -0.001 | -0.2695 | No |
| 55 | HAX1 |  |  | 3951 | -0.007 | -0.2881 | No |
| 56 | CYB5A |  |  | 3954 | -0.007 | -0.2878 | No |
| 57 | COL5A1 |  |  | 3991 | -0.009 | -0.2928 | No |
| 58 | CHPF2 |  |  | 4024 | -0.011 | -0.2971 | No |
| 59 | PGAM1 |  |  | 4056 | -0.013 | -0.3009 | No |
| 60 | B3GAT3 |  |  | 4115 | -0.017 | -0.3089 | No |
| 61 | ANKZF1 |  |  | 4141 | -0.018 | -0.3113 | No |
| 62 | VCAN |  |  | 4158 | -0.019 | -0.3121 | No |
| 63 | AKR1A1 |  |  | 4160 | -0.019 | -0.3105 | No |
| 64 | IDUA |  |  | 4172 | -0.020 | -0.3105 | No |
| 65 | PSMC4 |  |  | 4249 | -0.025 | -0.3206 | No |
| 66 | CHST12 |  |  | 4343 | -0.032 | -0.3328 | No |
| 67 | ISG20 |  |  | 4361 | -0.033 | -0.3325 | No |
| 68 | EXT2 |  |  | 4366 | -0.033 | -0.3302 | No |
| 69 | KIF2A |  |  | 4508 | -0.045 | -0.3490 | No |
| 70 | ADORA2B |  |  | 4561 | -0.049 | -0.3530 | No |
| 71 | PGM2 |  |  | 4642 | -0.055 | -0.3610 | No |
| 72 | SLC35A3 |  |  | 4705 | -0.060 | -0.3656 | No |
| 73 | GALE |  |  | 4750 | -0.064 | -0.3669 | Yes |
| 74 | P4HA1 |  |  | 4761 | -0.065 | -0.3626 | Yes |
| 75 | PLOD2 |  |  | 4764 | -0.065 | -0.3570 | Yes |
| 76 | SLC25A10 |  |  | 4807 | -0.068 | -0.3576 | Yes |
| 77 | ERO1A |  |  | 4850 | -0.072 | -0.3578 | Yes |
| 78 | ENO1 |  |  | 4868 | -0.073 | -0.3539 | Yes |
| 79 | TXN |  |  | 4891 | -0.074 | -0.3507 | Yes |
| 80 | POLR3K |  |  | 4904 | -0.075 | -0.3458 | Yes |
| 81 | PKM |  |  | 4921 | -0.077 | -0.3414 | Yes |
| 82 | PMM2 |  |  | 4931 | -0.077 | -0.3358 | Yes |
| 83 | TPBG |  |  | 4976 | -0.081 | -0.3356 | Yes |
| 84 | GMPPB |  |  | 5033 | -0.085 | -0.3369 | Yes |
| 85 | SRD5A3 |  |  | 5113 | -0.093 | -0.3412 | Yes |
| 86 | CLN6 |  |  | 5118 | -0.093 | -0.3334 | Yes |
| 87 | PAXIP1 |  |  | 5142 | -0.096 | -0.3284 | Yes |
| 88 | STC2 |  |  | 5150 | -0.097 | -0.3207 | Yes |
| 89 | B4GALT1 |  |  | 5170 | -0.099 | -0.3147 | Yes |
| 90 | KIF20A |  |  | 5260 | -0.107 | -0.3195 | Yes |
| 91 | EGFR |  |  | 5316 | -0.110 | -0.3183 | Yes |
| 92 | GPC1 |  |  | 5348 | -0.113 | -0.3130 | Yes |
| 93 | SAP30 |  |  | 5390 | -0.117 | -0.3090 | Yes |
| 94 | PFKP |  |  | 5471 | -0.128 | -0.3103 | Yes |
| 95 | FKBP4 |  |  | 5480 | -0.129 | -0.2998 | Yes |
| 96 | PYGB |  |  | 5809 | -0.185 | -0.3362 | Yes |
| 97 | MET |  |  | 5815 | -0.187 | -0.3200 | Yes |
| 98 | CENPA |  |  | 5960 | -0.226 | -0.3228 | Yes |
| 99 | STMN1 |  |  | 5993 | -0.236 | -0.3064 | Yes |
| 100 | SDC1 |  |  | 5998 | -0.237 | -0.2855 | Yes |
| 101 | CXCR4 |  |  | 6012 | -0.242 | -0.2654 | Yes |
| 102 | GUSB |  |  | 6024 | -0.247 | -0.2446 | Yes |
| 103 | AURKA |  |  | 6026 | -0.248 | -0.2221 | Yes |
| 104 | DEPDC1 |  |  | 6030 | -0.251 | -0.1997 | Yes |
| 105 | LDHA |  |  | 6057 | -0.272 | -0.1791 | Yes |
| 106 | HSPA5 |  |  | 6061 | -0.274 | -0.1545 | Yes |
| 107 | NT5E |  |  | 6064 | -0.277 | -0.1296 | Yes |
| 108 | HMMR |  |  | 6148 | -0.340 | -0.1121 | Yes |
| 109 | IGFBP3 |  |  | 6184 | -0.397 | -0.0816 | Yes |
| 110 | NASP |  |  | 6187 | -0.403 | -0.0450 | Yes |
| 111 | CDK1 |  |  | 6246 | -0.627 | 0.0028 | Yes |
Table: GSEA details [plain text format]

  

Fig 2: HALLMARK\_GLYCOLYSIS: Random ES distribution      
 Gene set null distribution of ES for **HALLMARK\_GLYCOLYSIS**
